# Bypassing drug resistance by triggering necroptosis: recent advances in mechanisms and its therapeutic exploitation in leukemia

**DOI:** 10.1186/s13046-018-0976-z

**Published:** 2018-12-12

**Authors:** Xianbo Huang, Feng Xiao, Yuan Li, Wenbin Qian, Wei Ding, Xiujin Ye

**Affiliations:** 1 Department of Hematology, the First Affiliated Hospital, College of Medicine, Zhejiang University, 79# Qingchun Road, Hangzhou, 310003 China; 2 Malignant Lymphoma Diagnosis and Therapy Center, the First Affiliated Hospital, College of Medicine, Zhejiang University, Hangzhou, 310003 China; 3 Department of Pathology, the First Affiliated Hospital, College of Medicine, Zhejiang University, 79# Qingchun Road, Hangzhou, 310003 China; 4 Institute of Hematology, the First Hospital of Jiaxing, Jiaxing, 314000 China

**Keywords:** Necroptosis, Leukemia, Apoptosis resistance, RIPK1, RIPK3, MLKL

## Abstract

Resistance to regulated cell death is one of the hallmarks of human cancers; it maintains cell survival and significantly limits the effectiveness of conventional drug therapy. Leukemia represents a class of hematologic malignancies that is characterized by dysregulation of cell death pathways and treatment-related resistance. As the majority of chemotherapeutic and targeted drugs kill leukemia cells by triggering apoptosis, the observed resistance indicates the need for novel therapeutic strategies to reactivate nonapoptotic cell death programs in refractory leukemia. Necroptosis is a regulated form of necrosis that is precisely modulated by intracellular signaling pathways and thus provides potential molecular targets for rational therapeutic intervention. Indeed, accumulating evidence indicates that many current antitumor agents can activate necroptotic pathways and thereby induce leukemia cell death. Elucidation of the complete regulatory mechanism of necroptosis is expected to accelerate the development of novel therapeutic strategies for overcoming apoptosis resistance in leukemia. Here, we review the latest research advances in the regulatory mechanisms of necroptosis and summarize the progression of necroptosis-based therapeutic strategies in leukemia.

## Background

A delicate balance between cell proliferation and death is essential for maintaining the normal physiological function of organisms. Dysregulation of regulated cell death (RCD) contributes to a number of human diseases, including cancer. During tumorigenesis, neoplastic cells become resistant to RCD, which results in unlimited cell growth and the acquisition of additional oncogenic mutations [1, 2]. Recently, induction of cell death has been considered the most important mechanism of various antitumor agents. Thus, targeting cell death signaling is an attractive strategy for developing novel anticancer therapies [3].

In recent years, major developments have been made in the identification and characterization of cell death programs, and various forms of RCD, including apoptosis, autophagy and necroptosis, have been discovered and evaluated. Apoptosis is the first identified and best-studied form of RCD, and analyses of this process have led to the development of multiple anticancer drugs that reactivate apoptosis to kill tumor cells, including leukemia cells [4, 5]. However, inducing apoptosis by various antitumor agents is often limited by therapeutic resistance due to the impairment or deficiency of apoptotic pathways [6]. Thus, identification of more thoughtful therapies that target alternative forms of RCD is the main focus in cancer research.

Necrosis was previously considered to be a random and passive process that required no specific molecular events. However, a regulated type of necrosis (so-called necroptosis) was recently discovered via identification of chemical inhibitors of necrotic cell death (necrostatins), which underlines its regulated nature [7, 8]. Receptor-interacting protein kinase 1 (RIPK1) is a critical regulator of necroptosis. RIPK3 acts as a downstream mediator of RIPK1 [9], and mixed lineage kinase domain-like (MLKL) is regarded as the key player in necroptosis execution [10].

Leukemia refers to a variety of malignant clonal diseases of hematopoietic stem cells that can induce death and is one of the top ten most dangerous causes of mortality for human beings [6]. In recent years, the survival rates of leukemia have significantly improved due to the development of individual chemotherapy and biological targeted therapy. However, the increasing rate of treatment-related resistance in leukemia remains a major challenge for researchers [11]. Given the rising significance of necroptosis in cancer, a better understanding of its detailed regulatory mechanisms is needed for the development of drugs to trigger necroptosis in leukemia cells, especially those with apoptosis resistance. A review of necroptosis and its relevance in leukemia is therefore urgently needed. In this review, we will discuss the regulatory mechanism of necroptosis in detail. We will also summarize the research progress made in induction of necroptosis in leukemia cells.

## Main text

### Mechanisms and regulation of necroptosis

#### Characteristics of necroptosis

**Fig. 1:**
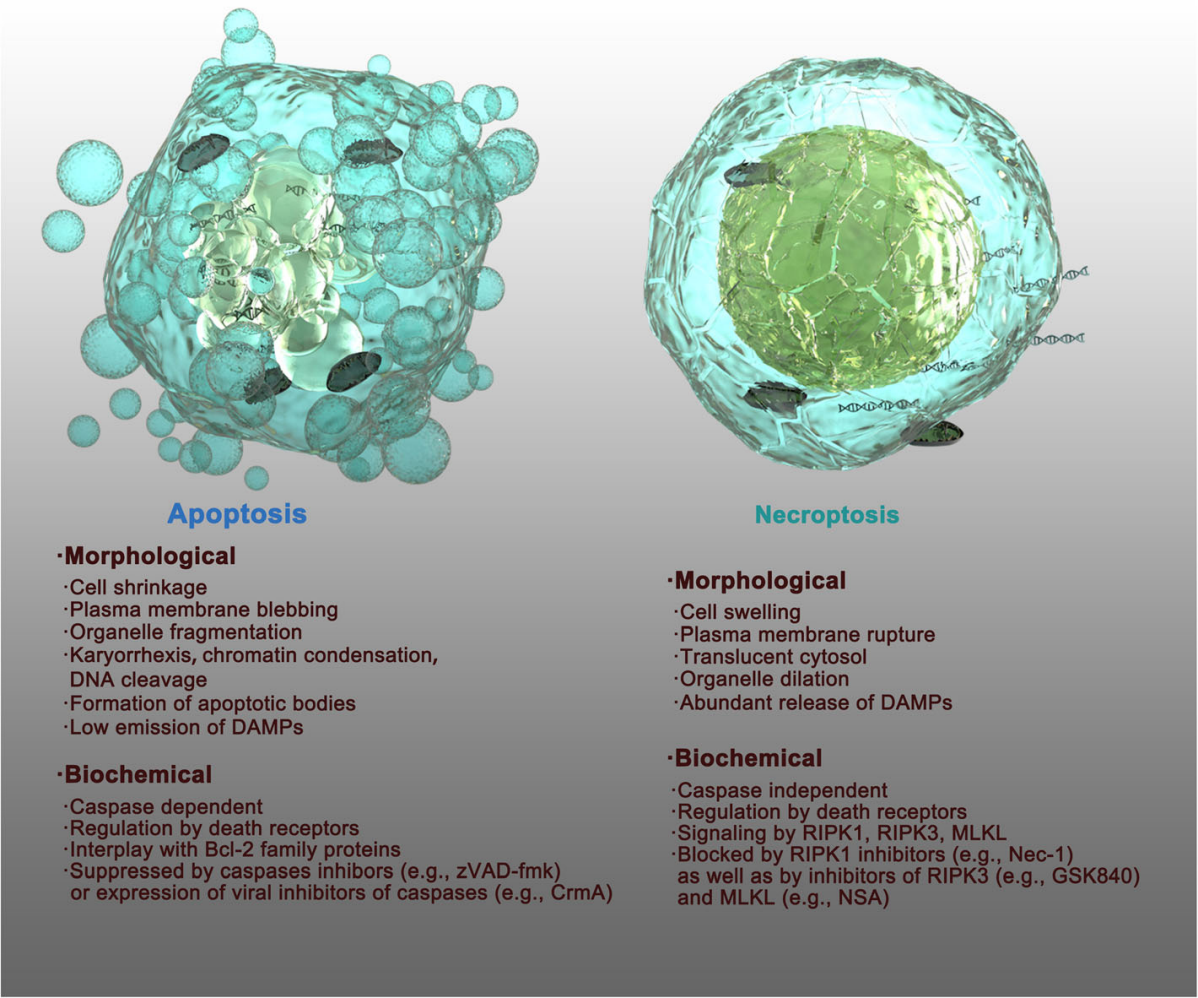
Schematic diagram describing the morphological and biochemical differences between apoptosis and necroptosis. Apoptotic cells are characterized by plasma membrane blebbing, cell shrinkage, organelle fragmentation, chromatin condensation, cleavage of chromosomal DNA and the formation of apoptotic bodies without rupture of the plasma membrane, and apoptotic cells show low emission of DAMPs. Necroptotic cells share some morphological features to apoptotic cells, resembling necrosis including cell swelling, plasma membrane rupture, translucent cytosol, and organelle dilation, and necroptotic cells are associated with the abundant release of DAMPs. At the biochemical level, apoptosis and necroptosis have different intracellular molecular mechanisms as described, and they can be specifically blocked by various types of inhibitors

Necroptosis is a novel characterized form of cell death that has several distinctive characteristics compared to other types of cell death, particularly apoptosis. Necroptosis is also called “programed necrosis” and shares some morphological features with necrosis, including early loss of plasma membrane integrity, translucent cytosol, increased cell volume and swollen organelles [9, 12]. Unlike necroptotic cells, apoptotic cells lack these features and are characterized by plasma membrane blebbing, cell shrinkage, chromatin condensation, cleavage of chromosomal DNA and formation of apoptotic bodies without rupture of the plasma membrane (Fig. 1) [13, 14]. At the biochemical level, apoptosis requires caspase activation and is mediated by the interplay of Bcl-2 family proteins or activation of death receptors. Apoptosis can be blocked by pan-caspase inhibitors (e.g., zVAD-fmk) or expression of viral inhibitors of caspases (e.g., CrmA) [13, 14]. Necroptosis is caspase-independent and controlled by RIPK1, RIPK3 and MLKL, which can be blocked by various specific small molecule inhibitors (Fig. 1) [7, 8, 15]. Another key feature of necroptotic cells is the release of damage-associated molecular patterns (DAMPs) and cytokines/chemokines due to the permeabilization of the plasma membrane, which can subsequently trigger robust inflammation and an immune response [16, 17]. In contrast, apoptotic cells and/or apoptotic bodies are engulfed and then dissolved via phagocytosis by antigen-presenting cells (APCs) or by neighboring cells [18], which do not typically induce a strong immune response (Fig. 1) [8].

Despite these distinctive features, the molecular mechanism of necroptosis is believed to be closely related to other forms of cell demise (e.g., apoptosis and autophagy) [19], which prompted us to explore the regulation and relative contributions of different cell death modes. Apoptosis and necroptosis share several upstream signaling elements [20]. Therefore, how does a cell decide whether to undergo apoptosis or necroptosis? Current views suggest that the choice of cell death is determined by a variety of factors, including stimuli, cell type, genetic background and the intracellular environment. Usually, apoptosis is the preferred mode of death for cells, and necroptosis functions as an alternative mechanism to eliminate stressed cells or infected cells that fail to undergo apoptosis [21]. However, necroptosis can also play a dominant role under certain circumstances, such as abnormal metabolism, genetic mutations, viral infection and exposure to some cytotoxic antitumor drugs [22–24]. More often, it is a continuous process from apoptosis to necroptosis [25, 26]. Intensified death signals and increased stress levels can switch cell death from apoptosis to necroptosis [27]. Autophagy is a lysosomal degradation system that engulfs the cytoplasm and organelles for cellular renovation and homeostasis, and it may also participate in crosstalk with necroptosis [19]. Sometimes, autophagy can serve as a scaffold or pivotal site to mediate the formation of necrosome complexes, which finally lead to MLKL phosphorylation and cell necroptosis stimulation [27, 28]. The interrelationship between necroptosis and other cell death pathways is complicated and should be further explored.

#### Triggers of necroptosis

Various stimuli can lead to the initiation of necroptosis [20]. Ligand-receptor interactions are extrinsic pathways for the initiation of necroptosis. Recent studies have shown that necroptosis can be induced by the engagement of death receptors (DRs) in the TNF superfamily, including TNF receptor-1 (TNFR1), FAS (also known as CD95 or APO-1), TNF-related apoptosis-inducing ligand receptor 1 (TRAILR1, also known as DR4), and TRAILR2 (also known as DR5, APO-2, TRICK or KILLER). These receptors trigger necroptosis via their common cytoplasmic death domains (DDs) [23, 29]. In addition to DRs, other types of stimuli, including engagement of Toll-like receptors 3 and 4 (TLR3, TLR4) by lipopolysaccharides (LPS), pathogen-derived double-stranded DNA/RNA (dsDNA/RNA), T-cell receptor stimulation, type I and type II interferons (IFNs), virus infection via the z-DNA sensor DNA-dependent activator of IFN regulatory factors (DAI) and genotoxic stress, can trigger necroptosis [23, 30–33]. Several other types of stimuli, including retinoic acid-inducible gene I (RIG-I), mitochondrial antiviral signaling protein (MAVS), DAMPs, protein kinase R (PKR) complexes, nucleotide-binding and oligomerization domain (NOD)-like receptors (NLRs) and some antitumor agents, also result in necroptosis [34, 35]. These triggers are considered to individually or jointly induce necroptosis in complicated physiological or pathological conditions. It is beyond the scope of this review to list all the stimuli related to necroptosis from the current literature; therefore, we summarize the above triggers, which are most likely important in necroptosis induction.

#### Initiation of necroptosis: necrosome formation

##### Canonical necrosomes

One of the most extensively studied and best-characterized signaling mechanisms of necroptosis is the binding of TNF-α to TNFR1, which subsequently recruits a series of intracellular proteins to form complexes involved in proinflammatory and survival signaling (complex I), apoptosis (complex II) and necroptosis (necrosome) [8, 36, 37]. Notably, apoptosis pathway inactivity or deficiency (e.g., when caspase-8 or apoptosis inhibitors [IAPs] are downregulated or inhibited) must prevail for TNFR1-mediated necroptosis to ensue [38].

**Fig. 2:**
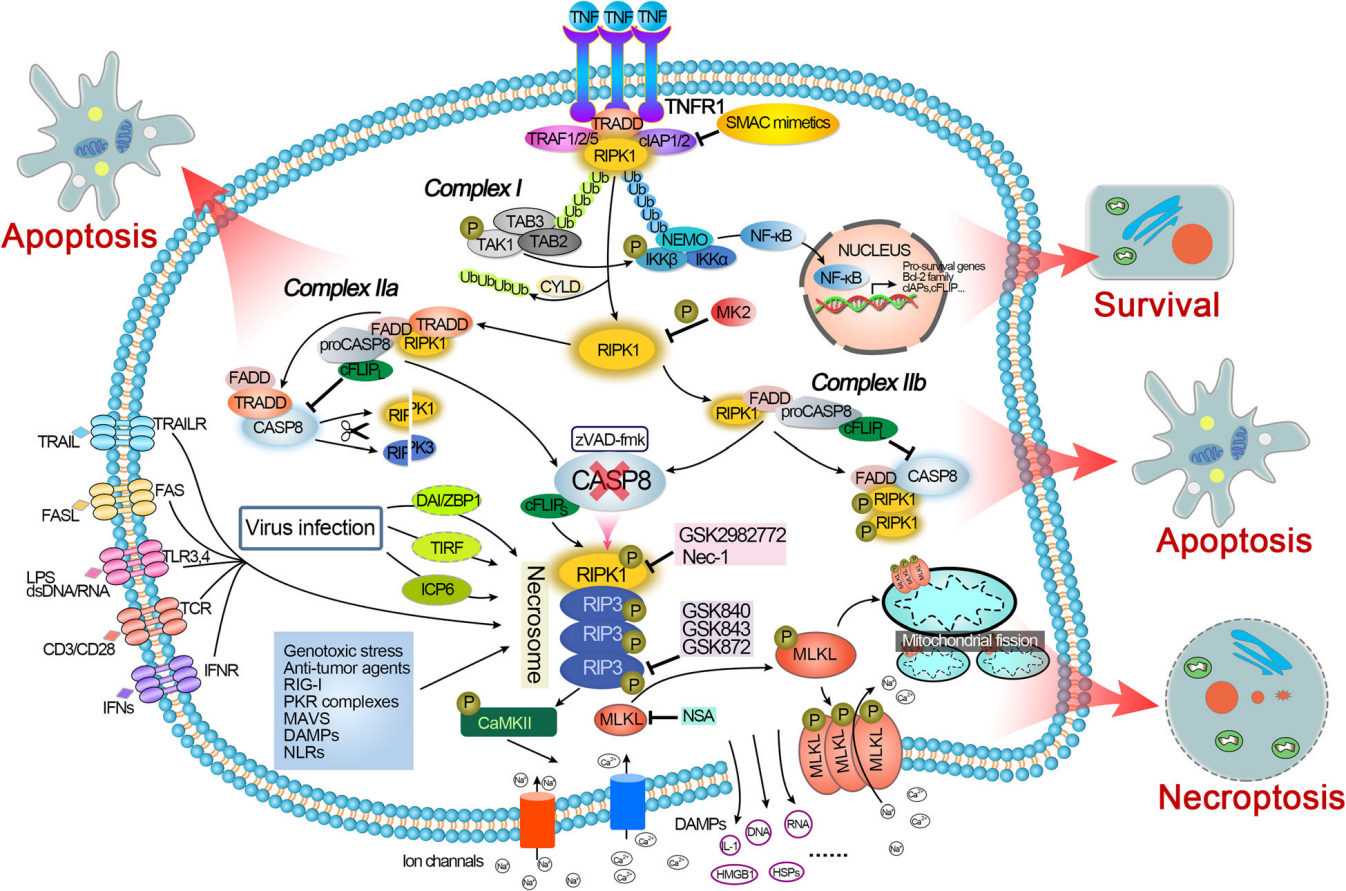
A schematic overview of the molecular signaling pathways involved in necroptosis. Upon TNF-α stimulation, activated TNFR1 recruits various downstream proteins, including RIPK1, to form prosurvival complex I, resulting in RIPK1 polyubiquitination and subsequently facilitating NF-κB signaling to prevent cell death (see text). Phosphorylation of RIPK1 by MK2 can also limit RIPK1 activation and the subsequent assembly of the death complex through the IKKα/β independent way. Inhibition of cIAPs (by Smac or Smac mimetics) leads to CYLD-mediated deubiquitination of RIPK1 and its dissociation from TNFR1, resulting in the formation of different prodeath complexes (complex IIa, IIb and the necrosome). Complex IIa contains TRADD and can be formed independently of the scaffold and kinase function of RIPK1. In contrast, complex IIb lacks TRADD and requires RIPK1 kinase activity for cell death induction. Complex IIa and IIb activate caspase-8, leading to apoptotic cell death. If caspase-8 activity is blocked, RIPK1 will bind to RIPK3 to form necrosomes and promote RIPK3 autophosphorylation and activation. Activated RIPK3 is currently known to function via at least two downstream effectors: MLKL and CaMKII, which are effector molecules leading to necroptosis through multiple mechanisms. Other stimuli, including FasL, TRAIL, CD3/CD28, LPS, dsDNA/RNA and IFNs, can stimulate their corresponding receptors to activate necrosomes to promote necroptosis. Infection with some viruses directly activates RIPK3 through DAI, TIRF or ICP6. Anticancer agents, genotoxic stress and some other factors can also trigger RIPK1/RIPK3-dependent necroptosis. Necroptosis is inhibited experimentally by specific inhibitors of RIPK1, RIPK3 and MLKL, as shown above

Under certain conditions, such as infection or tissue impairment, TNF-α binds to and stimulates TNFR1 through the preligand assembly domain of the extracellular portion of TNFR1 and then triggers its trimerization [39]. Upon activation, TNFR1 can recruit diverse intracellular proteins and induce the formation of a membrane-bound complex called complex I. Complex I consists of TNF-α receptor associated death domain (TRADD), E3 ubiquitin ligases TNF-α receptor associate factor 1, 2 and 5 (TRAF1, 2, 5), cellular inhibitor of apoptosis protein-1 and -2 (cIAP1/2) and RIPK1 (Fig. 2) [40–42]. In this complex, RIPK1 is polyubiquitinated by the ubiquitin ligase cIAP1/2 and other E3 ubiquitin ligases, and the polyubiquitin chain contributes to the recruitment of a number of proteins, such as transforming growth factor β-activated kinase 1 (TAK1), transforming growth factor β-activated kinase binding protein 2 and 3 (TAB2, 3), nuclear factor kappa B essential modulator (NEMO), and IkB kinase α/β (IKKα/β), and subsequently facilitates the nuclear factor κB (NF-κB) cell survival pathways [43–45] (Fig. 2). This change drives the expression of downstream proteins directly involved in apoptosis inhibition, such as B-cell lymphoma 2 (Bcl-2) family members, the caspase-8 inhibitor FLICE-like inhibitory proteins (cFLIP) and cIAPs [46–48]. cFLIP, a catalytically inactive homolog of caspase-8, was reported to be an important regulator of apoptosis and necroptosis [49]. The long cFLIP isoform (cFLIP_L_) binds to pro-caspase-8 and forms the caspase-8/cFLIP_L_ heterodimer (Fig. 2). For this reason, cFLIP_L_ reduces oligomerization of caspase-8 at FADD and finally inhibits apoptosis, but the caspase-8 still maintains sufficient proteolytic activity [50, 51]. Meanwhile, the heterodimer causes the cleavage of the necroptosis core regulators RIPK1 and RIPK3, thus inhibiting necroptosis [52, 53]. Therefore, the absence of cFLIP_L_ can induce caspase-dependent apoptosis or caspase-independent necroptosis. However, another short type of cFLIP isoform (cFLIP_S_) can combine with and inactivate caspase-8, which allows the activation of RIPK1/3 and thus leads to necroptosis (Fig. 2) [54]. Therefore, we believe that ubiquitylated RIPK1 can prevent cell death via activating survival pathways. Hence, complex I is a crucial checkpoint for cell survival and death. More recently, an additional transcription-independent checkpoint has been shown to modulate the contribution of RIPK1 to cell demise. RIPK1 phosphorylation by IKKα/β in complex I prevents RIPK1 kinase-dependent formation of the death complex [55]. RIPK1 is also a direct substrate of MAPK-activated protein kinase 2 (MK2). Phosphorylation of RIPK1 by MK2 can limit cytosolic activation of RIPK1 and the subsequent assembly of the death complex that drives RIPK1-dependent apoptosis and necroptosis, representing a mechanism that is distinct from the regulatory function of RIPK1 mediated by IKKα/β [56–58].

The degradation of cIAPs caused by second mitochondria-derived activator of caspases (Smac) or synthetic Smac-mimetics [47, 59–61] can reduce RIPK1 ubiquitination via deubiquitinase enzymes such as cylindromatosis (CYLD), resulting in RIPK1 dissociation from the plasma membrane and its conversion from a prosurvival into a pro-death protein [62, 63]. RIPK1 deubiquitination leads to the suppression of NF-κB and reduction of cFLIP and simultaneously promotes the formation of the cytosolic pro-cell death complex (complex II, also called ‘ripoptosome’) (Fig. 2) [54, 55]. Different types of complex II can be distinguished (IIa and IIb), depending on the composition and activity of the proteins therein. Complex IIa is formed after dissociation of TRADD from TNFR1 and results in the recruitment of downstream RIPK1, FAS-associated death domain protein (FADD) and pro-caspase-8, leading to caspase-8 activation. The activated caspase-8 then cleaves and inactivates RIPK1/RIPK3 and subsequently induces a type of RIPK1-independent apoptosis (Fig. 2) [8, 52, 54, 64, 65]. In conditions where cIAPs, TAK1, NEMO, and IKKα/β are inhibited or absent, a similar complex (complex IIb) is formed without TRADD (Fig. 2), where RIPK1 kinase activity is required for caspase-8 activation and promotes RIPK1 kinase activity-dependent apoptosis [66–69]. In some cell types or conditions, the levels of RIPK3 and MLKL are sufficiently high; caspase-8 activity is reduced, blocked or absent; and RIPK1 in complex II will recruit RIPK3. Then, a series of auto- and cross-phosphorylation reactions occur between RIPK1 and RIPK3 through their respective homotypic interaction motif (RHIM) domains, evolving to form a functional signaling complex called the necrosome [65, 70]. In necrosomes, activated RIPK3 recruits and phosphorylates the downstream pseudokinase MLKL, stimulating its oligomerization and translocation to the plasma membrane to trigger necroptosis (Fig. 2) [10, 71, 72]. The complex interaction between these cellular conditions forms the basis for either allowing or preventing the execution of necroptosis. The successful initiation of necroptosis via TNF-α/TNFR1 signaling is often based on the downregulation or inhibition of cIAPs and caspase-8 [72–74].

##### Noncanonical necrosomes

In classical necroptosis, necrosomes are formed via the RIPK1-RIPK3 activation model through the RHIM domain. Phosphorylation of RIPK1 and RIPK3 at

the kinase domain induces RHIM-mediated interactions, which result in the formation of amyloid-like filamentous signaling complexes [65, 70, 75] and culminate with necroptosis. In addition to RIPK1/3, other proteins such as TRIF (TIR-domain-containing adapter-inducing interferon-β; also known as TICAM1, TIR domain-containing adapter molecule 1), DAI (DNA activator of interferon; also known as ZBP1, Z-DNA binding protein 1) and ICP6 (viral ribonucleotide reductase large subunit) also have RHIM domains. These RHIM domain-containing proteins may function as a platform allowing RIPK3 oligomerization, autophosphorylation and activation through a RIPK1-independent mechanism that often involves an RHIM-RHIM interaction (Fig. 2) [30, 76–79]. Hence, they can form the necrosome, which is considered a noncanonical necrosome. For example, upon cytomegalovirus (CMV) infection in some cell types, DAI can activate RIPK3 directly via an RHIM-RHIM interaction but does not involve RIPK1 kinase activity [80]. After herpes simplex virus 1 (HSV-1) infection, the viral protein ICP6 interacts with RIP3 through a RHIM-RHIM interaction to trigger necroptosis and host defense, which do not require RIPK1 [79, 81]. Similarly, TLR3 and TLR4 initiate RIPK1-independent necroptosis mediated by the TRIF adaptor through the formation of the so-called TRIF-RIPK3 necrosome [30, 76]. Thus far, it is unclear how exactly RIPK3 is activated downstream of these RHIM domain-containing proteins. TRIF is an adapter that responds to the activation of TLRs, such as RIPK1 and RIPK3, and it is also a cleavage substrate for caspase-8. Recent studies have shown that inhibition of RIPK1 does not affect TLR3-mediated necroptosis. Unlike RIPK1, TRIF does not have kinase activity, indicating that the mechanism by which TRIF stimulates RIPK3 is different from the RIPK1-mediated RIPK3 activation [30]. Wang X et al. demonstrated that HSV-1 with an ICP6 deletion failed to induce effective necroptosis in infected cells. Furthermore, ectopic expression of ICP6, but not RHIM mutant ICP6, directly activated RIPK3/MLKL-mediated necroptosis [79]. Other studies have revealed that the perinatal lethality of RHIM-deficient RIPK1 knock-in mice can be rescued by DAI deficiency, which will prevent DAI/RIPK3/MLKL-dependent necroptosis during development. These findings indirectly proved that DAI will bind and activate RIPK3 to form a DAI-RIPK3 necrosome, which will participate in nonclassic necroptosis [82, 83].

#### Execution of necroptosis: MLKL activation

Recent studies have identified the pseudokinase MLKL as a major executioner of necroptosis [10]. Following stabilization of the RIPK1-RIPK3 complex, MLKL is recruited to form a functional necrosome [10, 72, 84]. Normally, MLKL remains inactive as a monomer in the cytosol [72]. Once the necrosome forms, the activated RIPK3 recruits and phosphorylates the downstream MLKL at Ser345, Ser347, Ser358 and Thr357 and the mouse MLKL at Ser352 and Thr349 within the MLKL activation loop [10, 72, 85], which results in an open conformational shift of MLKL and exposure of its four-helical bundle domain [10, 86]. Destabilization of the structure promotes MLKL oligomerization, resulting in the translocation of the MLKL oligomer from the cytosol to the plasma membranes (as well as to intracellular membranes), where it compromises the membrane integrity to promote necroptotic death (Fig. 2) [87–89]. Several hypotheses have been proposed to explain the mechanism of MLKL oligomer targeting to the cellular membrane and induction of cell death. Some have suggested that the MLKL oligomer can directly form a pore in the plasma membrane after binding to negatively charged phospholipids, subsequently causing necrotic membrane disruption. Lipids play a crucial role in MLKL membrane targeting. Phosphorylated MLKL forms an oligomer that can interact with phosphatidylinositol phosphates (PIPs, mostly including PI[5]P and PI[4,5]P_2_) on the inner surface of the plasma membrane through a low affinity site in its N-terminal bundle domain [88, 89]. This process may result in different modes of membrane permeabilization (including carpet, barrel stave and toroidal) [90]. Interestingly, necroptosis can be blocked by interfering with the formation of PI(5)P or PI(4,5)P_2_ [88]. The relocalization of MLKL oligomers to the plasma membrane also induces ion-pore dysregulation (including Na^+^ and Ca^2+^ influx) through association with ion channels, which accelerates membrane permeabilization and damage due to the increase in intracellular osmotic pressure and nanopore formation in the plasma membrane (Fig. 2) [91–94]. Alternatively, RIPK3 can activate Ca^2+^-calmodulin-dependent protein kinase II (CaMKII) independently of MLKL, which in turn induces an ion influx by activating multiple ion channels (Fig. 2) [95]. Nonetheless, it is still unclear whether the observed ion influx is a consequence or the cause of necroptotic cell death [76].

MLKL oligomers also target the mitochondrial membrane and induce mitochondrial permeability transition (MPT) alteration, which can subsequently cause mitochondrial disruption [96]. Mitochondrial disruption induces ATP depletion and excessive reactive oxygen species (ROS) production to contribute to cell death [97]. ROS are an important effector during necroptotic cell death and can kill cells in a positive feedback loop [12, 96, 98]. Although we have listed various execution mechanisms downstream of necrosomes, the full necroptotic cell death process remains to be elucidated.

#### Necroptosis and inflammation: DAMPs release

Necroptosis is closely associated with inflammation. The final stage of cell necroptosis, known as propagation, can lead to robust inflammation mainly through massive release of intracellular contents [17]. The majority of these cellular components are collectively described as DAMPs (Fig. 2) [99]. In contrast, apoptosis is generally nonimmunogenic because of plasma membrane shrinkage and orderly intracellular content disassembly, which results in nearly no release of DAMPs [16, 17]. DAMPs represent a collection of cellular components and molecules that are exposed or released by dying, injured or stressed cells, which act as a key contributor to triggering the inflammatory response. Generally, DAMPs include cytokines and alarmins that are released mainly by dying cells, such as the interleukin-1 family cytokines and S100 proteins. Additionally, several cellular components that are originally functional and nonimmunological can be released by damaged cells to act as DAMPs. These include histones and HMGB (high-mobility group protein) family members, DNA and RNA outside of nuclei or mitochondria, ribonucleoproteins, heat-shock proteins, purine metabolites, F-actin, calreticulin, etc. [17, 99, 100]. The release of DAMPs from the disintegrating cells suffering necroptosis is generally believed to be the primary mechanism of the inflammatory response mediated by MLKL-necrosome activation and MLKL oligomer insertion in the plasma membrane [17, 101]. This hypothesis has been supported by evidence that specific DAMPs are released by necroptotic cells, which are important mediators of inflammation [102]. These necroptosis-specific DAMPs include cytosolic lactate dehydrogenase and lysosomal hexosiminidase, as well as organ-specific proteins, such as heart or kidney creatine kinase and liver alanine aminotransferase [102]. Based on these findings, we speculate that necroptosis-specific DAMPs can be used for diagnostic biomarker development compared with other types of regulated necrotic cell death events, such as pyroptosis or ferroptosis [8]. To date, the full range of the specific DAMPs as mediators of necroptosis-induced inflammation requires further investigation.

#### Detection and pharmacological targeting of necroptosis

Due to a lack of specific molecular markers of necroptosis, a combination of approaches is often required to distinguish necroptosis from other cell death modalities. Transmission electron microscopy (TEM) or H&E staining is widely used to provide morphological evidence of necrosis [103]. PI permeability, loss of mitochondrial membrane potential (MMP), production of intracellular ROS, depletion of ATP and other factors are the detectable characteristics of necroptosis, but they do not distinguish necroptosis from other types of cell death [103, 104]. RIPK1, RIPK3 and MLKL are usually regarded as essential biochemical markers of necroptosis. Their activation can be detected by changes in the protein expression and phosphorylation status using immunoblotting or immunostaining [105, 106]. The formation of necrosome complexes can be observed by RIPK1/RIPK3 and RIPK3/MLKL interactions using immunoprecipitation or other methods [75]. The existence of RIPK1, RIPK3 and MLKL is necessary for necroptosis execution. We can use various approaches, such as gene knockout, siRNA/shRNA knockdown, small-molecule inhibitors and kinase-dead or interacting domain-deficient mutants, to further determine the role of these molecules in necroptosis. Researchers have made major efforts to develop small-molecule inhibitors that target these proteins (Fig. 1). Necrotatin-1 (Nec-1) was the first RIPK1 inhibitor identified by Yuan J’s group [7], and it has recently been widely used in the study of necroptosis. However, Nec-1 is not just the inhibitor of RIPK1 but also a potent inhibitor of indoleamine 2,3-dioxygenase (IDO), which is an immunomodulatory enzyme that regulates the formation of kynurenine [107]. Thus, interpretation of the results obtained with Nec-1 should always be used with caution. Additionally, GSK2982772 is a newly identified RIPK1 inhibitor detected by chemical screening [108]. The RIPK3 inhibitors GSK840, GSK843, GSK872 [30, 109] and dabrafenib [110] and the MLKL inhibitor necrosulfonamide (NSA) [72] are also used for research. In addition, the anticancer drugs ponatinib and pazopanib were recently found to inhibit both RIPK1 and RIPK3 (Fig. 2) [111]. Other types of RIPK1/RIPK3/MLKL inhibitors are still under development.

### Therapeutic induction of necroptosis in leukemia cells

Impairment of cell death pathways and evasion of RCD, especially apoptosis, are hallmarks of various cancers, including leukemia, that contribute to tumor initiation, progression and treatment resistance [1, 112]. Resistance to chemotherapy is currently a major problem in cancer treatment, and it is frequently associated with failure of tumor cells to undergo apoptosis [1]. Therefore, there is an urgent need to develop new therapies to promote cell death in cancers. Necroptosis, as a recently identified form of nonapoptotic RCD, may offer an alternative option to trigger apoptosis-resistant cancer cell death. Elucidation of the signal transduction pathways of necroptosis in cancer cells is expected to help develop novel strategies to trigger necroptosis in leukemia therapy. Thus far, accumulating work has proven that the induction of necroptosis may overcome drug resistance in cancers. In the following paragraphs, we provide a brief summary of findings regarding necroptosis in several major types of leukemia (Table 1).

**Table 1:** Necroptosis-inducing anti-leukemia agents

Disease	Agents	Targets	Mechanisms of necroptosis	Ref
AML	Birinapant+Emricasan	cIAPs, caspase-8	TNFR1 signaling; RIPK1/RIPK3/MLKL dependent	[123]
	BV6+zVAD-fmk	cIAPs, pan-caspase	RIPK1/RIPK3/MLKL dependent; autocrine TNF-α	[124]
	BV6+Cytarabine	cIAPs, DNA synthesis	RIPK1/RIPK3/MLKL dependent; autocrine TNF-α	[125]
	BV6+Azacitidine or Decitabine	cIAPs, DNA methylation	RIPK1/RIPK3/MLKL dependent; autocrine TNF-α	[126]
	BV6+MS275 or SAHA	cIAPs, Histone deacetylase	RIPK1/RIPK3/MLKL dependent; autocrine TNF-α	[127]
	HXR9	HOX/PBX dimer	RIPK1 dependent	[133]
	Diphtheria toxin GM-CSF	Protein synthesis	RIPK1 dependent	[137]
	Erastin	Unknown	RIPK3 dependent; c-JNK and p38 dependent	[138]
ALL	BV6+Dexamethasone	cIAPs, Glucocorticoid receptor	RIPK1/RIPK3/MLKL activation; Bak activation and mitochondrial perturbation	[143]
	BV6, LCL161, Birinapant	cIAPs	RIPK1/RIPK3/MLKL dependent; autocrine TNF-α; enhanced by hyperosmotic stress	[145]
	BV6 + Azacytidine	cIAPs, DNA methylation	RIPK1/RIPK3/MLKL-dependent; autocrine TNF-α	[145]
	Obatoclax	Bcl-2	Autophagy-dependent; mediated by RIPK1, CYLD	[149, 151]
	MG132, Bortezomib	Proteasome	RIPK3/MLKL dependent; accumulation of polyubiquitinated RIPK3	[154]
CLL	Ethacrynic acid	LEF1	CYLD activation	[159, 160]
CML	LQFM018	Unknown	TNFR1 and CYLD upregulation; involvement of dopamine D4 receptor	[165]
	Pig7	Lysosomal	MLKL activation; alteration of MMP and ROS levels	[167]

#### Acute myeloid leukemia

Acute myeloid leukemia (AML) is an aggressive disease that represents the most frequent malignant myeloid neoplasm in adults [113]. Despite current aggressive treatment strategies, the prognosis of AML is still poor due to its low survival and high relapse rate [113]. Thus far, most current therapies exert their antileukemic effects by promoting apoptosis in AML cells [114]. Apoptosis-resistant AML cells usually fail to undergo apoptosis due to the impairment of related pathways [114], and thus, induction of nonapoptotic cell death, such as necroptosis, is needed to overcome the treatment resistance and improve the outcomes of AML.

IAP proteins represent a family of antiapoptotic proteins that block RCD through various mechanisms [115]. As we described before, the IAP family members cIAP1/2 can act as E3 ubiquitin ligases that mediate ubiquitination of RIPK1 and contribute to canonical NF-kB signaling activation, which leads to cell survival [43]. Once deubiquitinated, RIPK1 can promote apoptosis or necroptosis based on the caspase-8 activity [40]. Another IAP, membrane X-linked inhibitor of apoptosis (XIAP), is known to block apoptosis by inhibiting caspase-9 and -3/-7 activation [116]. Therefore, the IAPs may be an important node that determines cell survival or death. IAPs can be neutralized by Smac, which is released from the mitochondrial intermembrane space into the cytosol during apoptosis [115]. Therefore, Smac can cause cell death via two pathways: a caspase-dependent apoptotic pathway or a caspase-independent necroptotic pathway. IAPs were shown to be overexpressed in AML cells and correlate with poor prognosis [117–119], so they are considered promising targets for therapeutic purposes. Smac mimetics have been artificially designed in recent years to antagonize IAP proteins [47, 48, 115, 120–122]. Thus, using Smac mimetics can induce necroptosis as an alternative option for AML cells that are refractory to apoptosis. [73]. Brumatti G et al. [123] found that AML cells are sensitive to clinical Smac mimetic birinapant-induced apoptosis. Blocking the activity of caspase-8 by the clinical caspase inhibitor emricasan/IDN-6556 can augment the killing effect of birinapant by triggering necroptotic cell death. The researchers finally demonstrated the antileukemic efficacy and safety of the induction of necroptosis via a birinapant/emricasan combination in vivo, which should be clinically investigated as a therapeutic opportunity. Another type of Smac mimetic, BV6, can also elicit necroptosis depending on TNF-α and the activation of its downstream components of the necroptosis pathway, such as RIPK1, RIPK3 and MLKL, in AML cells, in which apoptosis is inhibited pharmacologically by the pan-caspase inhibitor zVAD-fmk or genetically by caspase-8 knockdown. Additionally, BV6 triggers necroptosis in apoptosis-resistant patient-derived AML blasts [124]. Several studies have suggested that BV6 can act in concert with a series of commonly used clinical drugs in AML treatment, such as cytarabine, the demethylating agents azacitidine or decitabine and the histone deacetylase inhibitors MS275 or SAHA, to trigger necroptosis in apoptosis-resistant AML cells in a synergistic manner mediated by TNFα/RIPK1/RIPK3/MLKL activation [125–127]. Interestingly, the multitargeting kinase inhibitor sorafenib used for the treatment of AML [128] can limit BV6-induced necroptosis in apoptosis-resistant AML cells via inhibiting phosphorylation of MLKL, which has important implications for the application of sorafenib in treatment of AML [11]. Although admittedly still in early stages of development, some clinical studies with Smac mimetics have been performed in myeloid malignancies, including birinapant in AML (NCT01486784), myelodysplastic syndrome (NCT01828346, NCT02147873) and chronic myelomonocytic leukemia (NCT02147873). Additionally, there are/have been some clinic trials using Smac mimetics (e.g., birinapant, LCL161 and AT-406) in lymphoma (NCT00993239, NCT01078649) and multiple myeloma (NCT03111992). Evidence obtained indicate that these Smac mimetics exert favorable antitumor activity in treatment resistance patients including leukemia and was well tolerated. Vomiting, nausea, diarrhea and other gastrointestinal symptoms were common side effects of these drugs but not severe. Neutropenia and cytokines releasing were also observed in some patients, but they are controllable [129–131]. The data above indicated that Smac mimetics might be a novel effective clinical agent in treating drug-resistance leukemia by triggering necroptosis, and thus need to be further studied.

In addition to the Smac mimetic-centered strategy, other methods or mechanisms have also been demonstrated to induce necroptosis and thus bypass apoptosis resistance in AML cells. Alharbi R et al. found that blocking the interaction of HOX family transcription factors, which play key roles in AML cell survival [132], with the cofactor PBX by a short, cell-penetrating peptide (HXR9) can induce necroptosis in AML-derived cell lines and primary AML cells from patients [133]. Additionally, this effect can be synergistically enhanced by the protein kinase C signaling inhibitor Ro31 [133]. Granulocyte-macrophage colony-stimulating factor receptors (GM-CSFR) are overexpressed in most AML cells [134], which are responsive to GM-CSF [135]. Thus, selectively targeting cells with increased levels of GM-CSF receptors may be a promising method for more effectively treating AML. Several studies have shown that a recombinant fusion protein diphtheria toxin-GM-CSF (DT-GMCSF) exerts selective killing effects on AML cells by inducing apoptosis, while sparing normal hemopoietic cells [134, 136]. Horita H’s research showed that DT-GMCSF triggers necroptotic death in AML cells that are defective in apoptosis, suggesting that DT-GMCSF can activate multiple death pathways, including necroptosis and apoptosis [137]. In addition, the quinazolinone derivative erastin that exhibits synthetic lethality with expression of the RAS oncogene was recently shown to induce mixed types of cell death, including necroptosis, in AML cells. The erastin induced necroptosis is RIPK3 dependent manner and related to c-JUN N-terminal kinase (c-JNK) and p38 [138].

#### Acute lymphoblastic leukemia

Despite aggressive application of individualized chemotherapy, acute lymphoblastic leukemia (ALL) patients with high-risk, drug-refractory or relapsed disease still have a poor prognosis [139, 140]. As in many tumors, general deregulation of cell death pathways and failure to undergo chemotherapy-induced apoptosis constitute a key mechanism for drug resistance and clonal escape in ALL [141, 142]. This finding emphasizes the need to develop alternative strategies to induce other types of RCD, such as necroptosis, in ALL.

As mentioned above, Smac mimetic-based therapies are promising strategies to trigger necroptosis in apoptosis-resistant cells. The Smac mimetic BV6 and dexamethasone cooperate in the induction of necroptosis in ALL cells that are deficient in caspase-dependent apoptosis activation [143]. Furthermore. Rohde K et al. found that BV6/dexamethasone-triggered necroptosis relies on RIPK1/RIPK3/MLKL activation, followed by downstream Bak activation and mitochondrial perturbation (including ROS production and a drop in MMP), suggesting that mitochondrial dysfunction might serve as an amplification step in this process [143]. Using patient-derived xenograft models and CRISPR-based genome editing methodology, researchers demonstrated that another type of Smac mimetic, birinapant, can circumvent escape from apoptosis in drug-resistant and relapsed ALL by activating RIPK1/RIPK3/MLKL-dependent necroptosis [144]. Similar to its effects in AML, the Smac mimetic BV6 can also cooperate with the demethylating agent azacytidine to induce necroptotic cell death in ALL cells that are resistant to apoptosis [145]. Interestingly, hyperosmotic stress can boost Smac mimetic (e.g., BV6, LCL161, birinapant)-induced necroptosis by complementary TNF secretion in ALL cells, thus indicating that physicochemical modulation of the tumor environment can be utilized to enhance treatment efficacy of Smac mimetic-based therapies for ALL [146].

Antiapoptotic Bcl-2 protein family members (e.g., Mcl-1, Bcl-X_L_) are highly expressed in ALL and are often associated with chemotherapy resistance [147, 148]. Based on these findings, the potential of the pan-Bcl-2 family small molecule inhibitor obatoclax for combination therapy in refractory ALL was studied. Bonapace L et al. demonstrated that a combination of obatoclax could resensitize multidrug-resistant childhood ALL cells to glucocorticoids through rapid activation of autophagy-dependent necroptosis [149]. *MLL* gene translocations, which occur in 75% of ALL in infants younger than 1 year old, are related to poor prognosis [150]. Additionally, the expression of Bcl-2 family members is often upregulated in *MLL-translocation* infant ALL cells [151]. Urtishak K et al.’s study described multiple death mechanisms, including necroptosis, of obatoclax in killing infant ALL primary cells with *MLL* translocations that confer chemotherapy resistance [151]. Though the limited efficacy and significant toxicity of obatoclax in the recently clinic trials restrict its application in clinical therapy, obatoclax still has the potential as a cancer therapy when modified for less toxic side effects or when combined with other antileukemia agents [152]. Defects in the ubiquitin-proteasome system (UPS) can lead to various disorders, including tumorigenesis. Clinically targeting UPS has been proven to be an effective therapeutic approach in treating multiple cancers [153]. Moriwaki K et al. showed that treatment with the proteasome inhibitors MG132 and bortezomib can directly activate the necroptotic pathway in the ALL-derived cell line Jurkat, which is based on the RIPK3-MLKL interaction via RHIM domains [154].

#### Chronic lymphoblastic leukemia

Chronic lymphoblastic leukemia (CLL) refers to a hematological malignancy characterized by the clonal expansion and accumulation of small B lymphocytes that have a mature appearance [155]. Despite the substantial progress in pathobiology research and the development of effective treatment regimens, CLL remains incurable at present [156]. An impaired cell death program contributes to the accumulation of monoclonal B cells as well as chemotherapy resistance [157]. Recent studies have revealed that CLL cells have defects not only in the apoptosis program but also in the necroptosis pathway. Similar to other studies, researchers have observed the production of TNFα and degradation of cIAP1/2 in CLL cells treated with Smac mimetics. Unexpectedly, CLL cells are unable to form the ripoptosome complex and are killed by apoptosis or necroptosis, which may be associated with the aberrant upstream NF-kB regulation [158]. Li J’s team also found that CLL cells failed to undergo necroptosis upon TNF-α/zVAD-fmk costimulation due to the strong downregulation of RIPK3 and CYLD [159]. Then, the researchers found that the high level of Lymphoid enhancer-binding factor 1 (LEF1), a downstream effector of Wnt/β-catenin signaling, might act as a transcription repressor of CYLD and predict adverse prognosis (decreased TFS and OS) in CLL [159, 160]. Inhibiting LEF1 by ethacrynic acid or gene knockdown can sensitize CLL cells to death receptor ligation-induced necroptosis, which may be a promising therapeutic strategy for CLL [159, 160]. Venetoclax, a small and orally available molecule that specifically targets Bcl-2, was recently approved by the United States Food and Drug Administration for the treatment of CLL. Venetoclax showed a manageable safety profile and induced substantial responses in patients with relapsed CLL, including those with poor prognostic features, and venetoclax represents the most likely future direction in targeted CLL therapy [161]. However, the relationship between necroptosis stimulation and the killing effects of venetoclax on CLL cells remains unclear and needs to be further investigated.

#### Chronic myeloid leukemia

The introduction of selective BCR-ABL tyrosine kinase inhibitors (TKIs) has significantly improved the prognosis of chronic myeloid leukemia (CML), mainly through inducing apoptotic cell death, but drug resistance still exists in some patients [162]. TKI-resistant CML cells are usually characterized by apoptosis resistance [163, 164] and thus require an alternative approach, such as necroptosis, to reactivate cell death in CML. Unfortunately, limited progress has been made in studying necroptosis in CML, probably due to its favorable prognosis. Here, we provide a brief review of this progress. A newly synthesized piperazine-containing compound, LQFM018, has been proven to promote necroptosis in the CML cell line K562, as shown by the cell membrane rupture, mitochondrial damage with MMP loss and ROS overproduction and upregulation of TNFR1 and CYLD, with no involvement of caspase-3 and caspase-8 activation. This process most likely involves the dopamine D4 receptor [165]. The p53-induced gene 7 (pig7), which localizes to the lysosomal membrane, is considered one of the key factors involved in p53-induced apoptosis [166]. Liu J and his colleagues’ work has shown that overexpression of pig7 did not directly activate the caspase apoptotic pathway but decreased the lysosomal stability

and significantly sensitized the drug-resistant CML cell line K562/ADM (has low endogenous pig7 expression) to chemotherapeutic drugs through necroptosis

involving multiple cell death mechanisms. This cell death is associated with alteration of MMP and ROS levels, as well as MLKL activation [167]. In addition, homoharringtonine (HHT), a plant alkaloid that was recently approved by the FDA to treat patients with CML, is regarded as an efficient sensitizer for TRAIL-induced necroptosis in multiple human solid tumor cell lines [168]. Based on this finding, HHT/TRAIL combination therapy may be used to treat apoptosis-resistant CML, which needs to be further studied and confirmed.

## Conclusions

Necroptosis has recently attracted attention as a form of RCD that can be triggered even under conditions of disabled apoptosis. Notably, activation of the RIP1/RIP3/MLKL pathway was shown to be the main mechanism for necroptosis initiation and execution. Because apoptosis evasion represents a hallmark of human cancers, including leukemia, therapeutic induction of necroptosis may open new directions for treatment strategies in apoptosis-resistant leukemia. While a series of drugs and compounds have been shown to trigger necroptosis in leukemia cells, the precise molecular targets of most of these agents in promoting leukocyte necroptosis remain unclear. Additionally, evidence has shown that some components of the cell death pathway that mediate necroptosis are often scarce or even lacking, which prompted us to obtain a deeper understanding of the molecular signaling network that regulates necroptotic cell death. In conclusion, targeting necroptosis for the treatment of leukemia presents significant advantages over current strategies. However, a better understanding of the underlying molecular mechanisms of necroptosis is required before necroptosis can be used in clinical therapeutic interventions.

## Abbreviations

ALL: Acute lymphoblastic leukemia
AML: Acute myeloid leukemia
APC: Antigen presenting cell
Bcl-2: B-cell lymphoma 2
CaMKII: Ca^2+^-calmodulin-dependent protein kinase II
CASP8: Caspase-8
cFLIP: FLICE-like inhibitory proteins
cFLIPL/S: Long/short type of cFLIP isoform
cIAP1/2: Cellular inhibitor of apoptosis protein 1, 2
c-JNK: c-JUN N-terminal kinase
CLL: Chronic lymphoblastic leukemia
CML: Chronic myeloid leukemia
CMV: Cytomegalovirus
CYLD: Cylindromatosis
DAI: DNA activator of interferon
DAMPs: Damage-associated molecular patterns
DDs: Death domains
DRs: Death receptors
dsDNA/RNA: Double strand DNA/RNA
DT-GMCSF: Diphtheria toxin GM-CSF
FADD: Fas-associated death domain protein
FASL: FAS ligand
GM-CSF: Granulocyte-macrophage colony-stimulating factor
GM-CSFR: GM-CSF receptor
HHT: Homoharringtonine
HMGB: High-mobility group protein
HSP: Heat-shock proteins
HSV-1: Herpes simplex virus 1
ICP6: Viral ribonucleotide reductase large subunit
IDO: Indoleamine 2,3-dioxygenase
IFNR: Interferon receptor
IFNs: Interferons
IKKα/β: IκB kinase α/β
IL-1: Interleukin-1
LEF1: Lymphoid enhancer-binding factor 1
LPS: Lipopolysaccharide
MAVS: Mitochondrial antiviral signaling protein
MK2: MAPK-activated protein kinase 2
MLKL: Mixed lineage kinase domain-like
MMP: Mitochondrial membrane potential
MPT: Mitochondrial permeability transition
Nec-1: Necrostatin-1
NEMO: Nuclear factor kappa B essential modulator
NF-κB: Nuclear factor κB
NLRs: NOD-like receptors
NOD: Nucleotide-binding and oligomerization domain
NSA: Necrosulfonamide
PKR: Protein kinase R
RCD: Regulated cell death
RHIM: Respective homotypic interaction motif
RIG-I: Retinoic acid-inducible gene I
RIPK1, 3: Receptor-interacting protein kinase 1, 3
ROS: Reactive oxygen species
Smac: Second mitochondria-derived activator of caspases
TAB2, 3: Transforming growth factor β-activated kinase binding protein 2, 3
TAK1: Transforming growth factor β-activated kinase 1
TCR: T-cell receptor
TEM: Transmission electron microscopy
TICAM1: TIR domain-containing adapter molecule 1
TKIs: Tyrosine kinase inhibitors
TLR3, 4: Toll-like receptors 3, 4
TNFR1: TNF receptor 1
TNF-α: Tumor necrosis factor α
TRADD: TNF-α receptor associated death domain
TRAF2, 5: TNF-α receptor associate factor 2, 5
TRAIL: TNF-related apoptosis-inducing ligand
TRAILR: TRAIL receptor
TRIF: TIR-domain-containing adapter-inducing interferon-β
Ub: Ubiquitin
UPS: Ubiquitin-proteasome system
XIAP: X-linked inhibitor of apoptosis
ZBP1: Z-DNA binding protein 1
